# Supplementary material for: Nanoscale real-time detection of quantum vortices at millikelvin temperatures
Source: Nat Commun. 2021 May 11;12:2645. doi: 10.1038/s41467-021-22909-3 (PMC8113507; doi:10.1038/s41467-021-22909-3)
Supplement: Supplementary file 1 — Supplementary information [file 41467_2021_22909_MOESM1_ESM.pdf]

Supplementary information for: Nanoscale Real-Time Detection of Quantum Vortices at Millikelvin Temperatures

A. Guthrie[1], S. Kafanov[1],* M. T. Noble[1], Yu. A. Pashkin[1], G. R. Pickett[1], V. Tsepelin[1], A. A. Dorofeev[2, 3], V. A. Krupenin[2, 3], and D. E. Presnov[2, 3, 4]
[1]*Department of Physics, Lancaster University, Lancaster, LA1 4YB, U. K.*
[2]*Quantum Technology Centre, Moscow State University, Moscow, 119991, Russia*
[3]*Faculty of Physics, Moscow State University, Moscow, 119991, Russia and*
[4]*Institute of Nuclear Physics, Moscow State University, Moscow, 119991, Russia*

TENSION OF THE BEAM IN VACUUM

The resonance frequencies of the beam can be modelled as the harmonics of a doubly clamped resonator:[1]

$$f_n = \frac{k_n^2}{\pi\sqrt{48}}\frac{w}{l^2}\sqrt{\frac{E}{\rho_{\mathrm{Al}}}}\sqrt{1+\gamma_n\left(\frac{l}{w}\right)^2\frac{T_0}{wtE}}, \qquad (1)$$

where η is the strain and w and l represent the width and length of the beam, respectively. The coefficients k_n and γ_n have different values depending on the eigenmode of the resonance: $k_1 = 4.7300$, $\gamma_1 = 0.2949$, $k_2 = 7.8532$, $\gamma_2 = 0.1453$, and $k_{n\geqslant 3} = \pi(n + 1/2)$, $\gamma_{n\geqslant 3} = 12(k_n - 2)/k_n^3$.

During fabrication, the Si_3N_4 layer is pre-stressed to improve the mechanical properties of the beam. Using the measured value of $f_0 = 2.166\,\mathrm{MHz}$, with Young's modulus $E = 70\,\mathrm{GPa}$, we can estimate the intrinsic nanobeam tension using Eq. (1) to be $T_0 = 5.6\,\mu\mathrm{N}$.

THE HYDRODYNAMIC SHIFT OF THE BEAM FREQUENCY IN LIQUID ^{4}HE

In liquid ^{4}He the nanobeam fundamental frequency will be shifted due to the displacement of fluid by the beam. At 10 mK the normal-component density is negligible, and we can thus ignore hydrodynamic damping. The hydrodynamic displacement can be modelled as an increase in the effective mass of the beam, which thus shifts the resonance frequency from the vacuum state value:[2]

$$\left(\frac{f_0}{f_H}\right)^2 = 1 + \beta\frac{\rho_H}{\rho_b} \qquad (2)$$

where ρ_H is the density of helium and β geometric constant. The resonance frequency for our beam in liquid helium at 10 mK is shifted by 50 kHz from the vacuum value to $f_H = 2.116\,\mathrm{MHz}$. The geometric constant can therefore be calculated as $\beta = 0.46$.

ACOUSTIC DAMPING

Acoustic damping is a frequency-dependent damping source for oscillators active at all temperatures. However, since the magnetomotive damping of the beam at 5 T is an order of magnitude higher than for acoustic damping,[3] we can neglect its affects here.

EFFECTS OF THE INTERACTION BETWEEN THE NANOBEAM, THE TRAPPED VORTEX AND THE SUBSTRATE

In order to minimise its energy, the trapped vortex will align its core along the nanobeam. The presence of a trapped vortex along the length of the nanobeam gives rise to a force which acts to increase the frequency of the nanobeam.

This arises from the interaction of the trapped vortex and its image in the substrate situated at a distance d from the beam. Using the method of images, we can calculate the interaction between the vortex and substrate, in other words, we remove the surface from the problem and assume that the trapped vortex interacts with a parallel image vortex located a distance $2d$ from it.

The interaction force per unit length between two vortices is given by:

$$\boldsymbol{f} = \boldsymbol{j}\times\boldsymbol{\kappa},$$

where $|\boldsymbol{\kappa}| = h/m_{^4\mathrm{He}} = 9.92\times10^{-8}\,\mathrm{m^2\,s^{-1}}$ is the ^{4}He circulation quantum; $|\boldsymbol{j}| = \rho_{^4\mathrm{He}}v_s$ is the flow density created by the image vortex at the position of the beam; the linear velocity of the superfluid at a distance r from the vortex core is given by $v_s = \kappa/(2\pi r)$.

The final expression for the attractive force between the vortex trapped on the beam and silicon surface is given by

$$|\boldsymbol{F}| = \frac{1}{4\pi}\frac{l}{d}\kappa^2\rho_{^4\mathrm{He}}.$$

Substituting our experimental parameters, we find the force per unit of length and the total attractive force to be:

$$|\boldsymbol{f}| = 115\,\mathrm{nN\,m^{-1}}; \qquad |\boldsymbol{F}| = 8.04\,\mathrm{pN}.$$

Under the action of this force, the beam will sag. Taking as origin one of the clamped ends of the nanobeam, the sag can be described by the function:

$$z(x) = \frac{1}{2}\frac{|\boldsymbol{f}|}{E}\left(\frac{x}{t}\right)^2\frac{(l-x)^2}{tw},$$

The maximum displacement at the centre of the beam is:

$$z_{\max}\left(\frac{l}{2}\right) = \frac{1}{32}\frac{|\boldsymbol{f}|}{E}\frac{l^4}{wt^3}.$$

In our case the maximum sag will be $z_{\max} = 0.734\,\mathrm{pm}$.

The maximum tension will be at $x = 0$ and $x = l$:

$$T_{\max} = \frac{1}{2}|\boldsymbol{f}|\frac{l^2}{t},$$

in our case giving $T_{\max} = 6.51\,\mathrm{nN}$. The total tension acting on the nanobeam is now given by $T_{\mathrm{tot}} = T_0 + T_{\max}$. Using T_{tot} as the value of the tension, the expected frequency due to a trapped vortex is found by substituting the result of Eq. (1) into Eq. (2) as

$$f_v = 2.117\,\mathrm{MHz}, \tag{3}$$

corresponding to a frequency shift of $f_v - f_H = 1\,\mathrm{kHz}$ which is comparable to what is observed.

* sergey.kafanov@lancaster.ac.uk

[1] Bao, M. *Analysis and Design Principles of MEMS Devices* (Elsevier Science, 2005).

[2] Bradley, D. I. *et al.* Operating nanobeams in a quantum fluid. *Sci. Rep.* **7**, 4876 (2017).

[3] Guénault, A. M. *et al.* Probing superfluid ^{4}He with high-frequency nanomechanical resonators down to millikelvin temperatures. *Phys. Rev. B* **100**, 020506 (2019).
